# *Ayekoo*! — Well done!

**DOI:** 10.1172/JCI158278

**Published:** 2022-02-01

**Authors:** Rexford S. Ahima

## Abstract

As the curtain draws on the 5-year term of the *JCI* editorial board at Johns Hopkins, I am filled with gratitude and would like to extend a warm *ayekoo* (Ghanaian salutation meaning “well done”) to our editors, staff, reviewers, and scientists for supporting the Journal. I am delighted to welcome the next *JCI* Editor in Chief, Elizabeth McNally — the first woman to lead the *JCI* since it was founded almost a century ago — and her team from Northwestern University.

Every 5 years since its inception in 1924, the *JCI* goes through a comprehensive process of selecting a new editor in chief and editorial board of active scientists, mostly ASCI members. This transition provides an opportunity for fresh ideas for publishing cutting-edge discoveries in an increasingly competitive environment of journal publishing. When Gordon Tomaselli was selected as the *JCI* editor in chief in 2017 (1), I agreed to serve as a deputy editor together with Arturo Casadevall, because I knew the *JCI* landscape very well, having been an associate editor when the board was at the University of Pennsylvania a decade ago. Moreover, I had just moved from Penn to Johns Hopkins and was excited to meet and work with colleagues across various departments. Gordon had superb institutional insight and was able to put together a team of esteemed associate editors mainly from Johns Hopkins and some from the University of Maryland and the National Institutes of Health.

The *JCI* board at Johns Hopkins was larger than previous boards and well positioned to meet a growing interest in biomedical research, especially oncology, neuroscience, infectious diseases, immunology, pulmonology, metabolism, vascular biology, gastroenterology, and nephrology. As a board, we strived to publish the highest-quality basic science and clinical research and build upon the strengths of the *JCI*. Our editorial board emphasized clinical relevance for original research articles, which focus on mechanistic insights into disease pathogenesis, diagnosis, and treatment. We also expanded the number of Clinical Medicine papers, which highlight advances in disease diagnosis, early-phase clinical trials, novel therapies, or observational studies that impact medical practice. In addition to publishing reviews, review series, commentaries, and editorials, our board also started a new Viewpoint platform for discussing issues important to biomedical research and healthcare (1). Moreover, to develop interest in the ASCI, *JCI*, and scientific editorial process, we established the *JCI* Scholar Program. A small number of MD-PhD students and fellows were selected to shadow associate editors and participate in the manuscript evaluation and editorial board discussion (1).

After serving as the editor in chief for a year, Gordon Tomaselli stepped down to become the dean of the Albert Einstein College of Medicine. Subsequently, I was elected to replace him (2). I kept the editorial board intact, with the only modifications being that Gordon Tomaselli and Gregg Semenza became deputy editors, with Arturo Casadevall continuing in his role as a deputy editor. Under my leadership, we maintained the editorial process started by Gordon, strived to assign manuscripts to the best-qualified handling associate editors, sought fair reviews, met weekly to discuss submissions, and worked closely with our staff editors and production team to expedite publications. As fate would have it, there was a global crisis ahead that would threaten our operations. In 2020, when the COVID-19 pandemic hit the United States, we had to make critical adjustments to the editorial process of the *JCI* (3). Much of the burden fell on our colleagues in infectious diseases, public health, pulmonology, and critical care who also had to prioritize their efforts to patient care. These specialists and other board members handled a huge submission of COVID-19 manuscripts (4). We converted our weekly roundtable editorial board meetings into video meetings, asked our editors and peer reviewers to guard against unreasonable revision requests if these experiments did not change the conclusions, and in cases where additional studies were required, we allowed authors more time to conduct essential experiments (4).

I am pleased to report that our board has built on the success of the Duke-UNC board and done well in the midst of the COVID-19 crisis (4). We have handled over 23,000 manuscripts, including a massive surge in 2020 that was driven partly by research into COVID-19 as well as pandemic-related lockdowns (4). We have published 1441 original research papers, 116 concise communications, 200 reviews, including 19 review series, and 374 commentaries. I am proud that we were able to expand the clinical medicine category by encouraging submissions addressing disease pathogenesis, diagnosis, and therapeutic interventions in human subjects. We have published 170 clinical medicine papers, many of which have been highly cited. Our Viewpoint initiative focusing on a range of topics of interest to the biomedical community and general readers is popular. The range of topics include climate change, race, diversity and health equity, physician-scientist training and mentorship, research collaboration, research funding, academia-industry collaboration, rising medication costs, novel therapies, etc. The 105 viewpoint articles that we published have so far garnered 1,187,517 views and 182,052 downloads. We hope the Viewpoint category will continue to connect the *JCI* to scientists, clinicians, and the public.

The *JCI* has played a prominent role in addressing rising trends of data error and manipulation (5). We screen images, require authors to provide detailed experimental methods, reagents, and resources, and to show primary data points on graphs. Until 2021, our image screening was done manually. I am pleased to report that we have started automated screening of submitted images using Proofig software. Image anomalies identified in manuscripts under consideration are then verified manually, discussed by the handling editors, and queries are sent to the authors. Depending on the circumstances, manuscripts may be rejected if the findings are no longer deemed credible or cannot be corrected, avoiding embarrassing mistakes for our authors. Although still a time-consuming process, we are excited that this automated screening tool enhances the journal’s ability to detect problems prior to publication and ensure scientific rigor and integrity.

The *JCI* was the first biomedical journal to launch “open publishing” in 1996, by making all research articles freely accessible to users online (6). The journal later implemented an online subscription for review articles and commentaries, a hybrid arrangement that was intended to promote the sharing of research discoveries while generating funds to support the ASCI’s activities (7). Recently, various funders of research programs have required journals to make all published papers freely available to users. In response to this mandate, I am happy to report that the *JCI* has successfully transitioned to “Gold Open Access” status. Starting from January 4, 2022, all published content in the *JCI* is available without charge to individual users and institutions. Authors of published articles retain copyright to their work, which is published with a Creative Commons Attribution License (CC BY 4.0). Users of our published articles are allowed to read, download, copy, distribute, print, search, and link to full journal texts, and use material for any lawful purposes, without seeking prior permission from the author or publisher (ASCI) so long as they appropriately cite the original publication. Thus, the *JCI* fully meets cOAlition S requirements for authors who receive funding from “Plan S signatories,” and we are fully compliant with open-access policies of the Howard Hughes Medical Institute, Wellcome Trust, and UK Research and Innovation, among others.

In addition to enhancing our open-access policy, the *JCI* has adapted to increasing demands for more immediate access to scientific results. Under our watch, the journal began considering manuscripts posted on preprint servers in 2018, and at the same time gave our authors the option of rapidly publishing the accepted manuscript in the In Press Preview format. These changes are critical to our responsiveness, and became all the more important during the COVID-19 pandemic. To better address immediate issues that may arise after publication, in June of 2021 we instituted a Letter to the Editor category. The bar for the published letters is very high — the critiques regarding methodology, results, or data interpretation must be substantive, and the authors of published papers being critiqued are given an opportunity to respond. To date, we have published 11 articles in the Letter to the Editor category.

It has been a privilege to work closely with Kathleen Collins, editor in chief of our sister journal, the *JCI Insight*. Seven hundred eighty-four manuscripts that did not meet the *JCI’*s publication priority but were viewed as high-quality research were transferred to and published in *JCI Insight*. I am pleased that many of the transferred papers were successful. We expanded our guaranteed review option, in which authors can designate a manuscript for external peer review and bypass initial editorial rejection, to include frequent reviewers for the journal in addition to ASCI members (8). This option is a token of our thanks for the generous efforts of our reviewers on which the *JCI* relies. We believe this program benefits our scientific community, while also giving the editorial board full control of the priority for publications, thereby avoiding some of the controversies that have plagued special member tracks at other society journals. We also continued the Conversations with Giants series, edited by Ushma Neill, with 18 interviews of pioneering researchers in biomedical science. In commemoration of the 100th anniversary of the discovery of insulin, several leaders in diabetes, i.e., Jesse Roth, C. Ronald Kahn, Jeffrey Flier, Barbara Kahn, and Daniel Drucker, were interviewed (9–13). These video series ran in tandem with a review series highlighting the history of insulin’s discovery, classification of diabetes, pathophysiology of glucose metabolism, and dietary and drug therapy of diabetes.

The ASCI and *JCI* are both striving to diversify their leadership and programs (14). One area of concern in journal publication is gender bias (15). Because of a shift toward collaborative research, we have seen a preponderance of papers designating two or more co–first authors, accounting for approximately one-third of our total publications. Unfortunately, some studies suggest that men are often named as first authors (16). To reduce bias in assigning authorship rank, the *JCI* instituted a requirement for corresponding authors to explain how the first-author position of contributing authors is determined (17). We believe it helps all co–first authors, regardless of gender, to have a clear statement of their contribution and the factors that determined authorship. We hope that these requirements will spur more conversations about how author order is determined and help promote equity in future projects.

Another highlight of our editorial board has been the *JCI* Scholar Program, which provided an opportunity for trainees to be mentored by associate editors, review manuscripts, and participate in editorial board meetings. Although we were only able to host a limited number of scholars, 22 MD-PhD students and postdoctoral fellows during our 5-year term, the feedback has been tremendous. Indeed, some of our scholars have written about their experiences in the *JCI* Viewpoint series (17, 18). I hope the *JCI* Scholar Program can be expanded in the future across many institutions.

The *JCI* takes great pride in its editorial leadership, which consists of academicians actively engaged in research and healthcare. Having a team of editors actively engaged in research undoubtedly raises the quality of scholarship and ensures our authors work with peers at all levels of the editorial process. However, I should emphasize that the success of the *JCI* is also dependent on a team of experienced staff editors who manage the day-to-day operations and transition of the board from one group to another. Our editorial board is heavily indebted to Sarah Jackson, Executive Editor, and her team of science editors — Corinne Williams, Elyse Dankoski, and Lisa Conti — for assisting us in implementing our vision for the *JCI*. Also, we are grateful to John Hawley and his ASCI executive staff, and to the *JCI’s* production staff for providing outstanding support. Many thanks to all of you for your dedication, professionalism, and friendship.

Finally, I am most honored to have had the opportunity to work with an exceptional team of editors and advisors. In my opinion (biased, of course), the Johns Hopkins *JCI* board had the deepest bench of biomedical researchers and academic leaders ever assembled. We had a dean of a medical school (Gordon Tomaselli); Nobel laureate, Lasker awardee, and member of the National Academy of Sciences (Gregg Semenza); department chairs (Mark Anderson, William Nelson, Arturo Casadevall, and Marsha Wills-Karp); several directors of divisions, institutes, centers and laboratories; and 9 members of the National Academy of Medicine (Gregg Semenza, Arturo Casadevall, Andy Feinberg, Thomas Quinn, Mark Anderson, Elizabeth Jaffe, Rex Ahima, Mariana Kaplan, and Ted Dawson). For me, working with our board was a daily revelation of knowledge, teamwork, and tireless desire to serve the scientific community. I will surely miss all of you.

In bidding farewell, I would like to share some *adinkra* symbols from my birth country, Ghana, that capture the essence of our *JCI* editorial team (Figure 1). *Adinkra* (meaning “farewell” or “good bye” in the Asante Twi language) symbols are printed on cloth to express societal beliefs, values, and philosophy. I have selected four symbols in celebration of our board: (a) one who does not know can know by learning; (b) wisdom, ingenuity, intelligence; (c) interdependence, cooperation; and (d) vigilance, preparedness for action. It’s been a fantastic journey. We have been honored by the trust granted to us by the ASCI to serve its membership and the greater scientific community. We wish great success to Elizabeth McNally and her team.

## Figures and Tables

**Figure 1:**
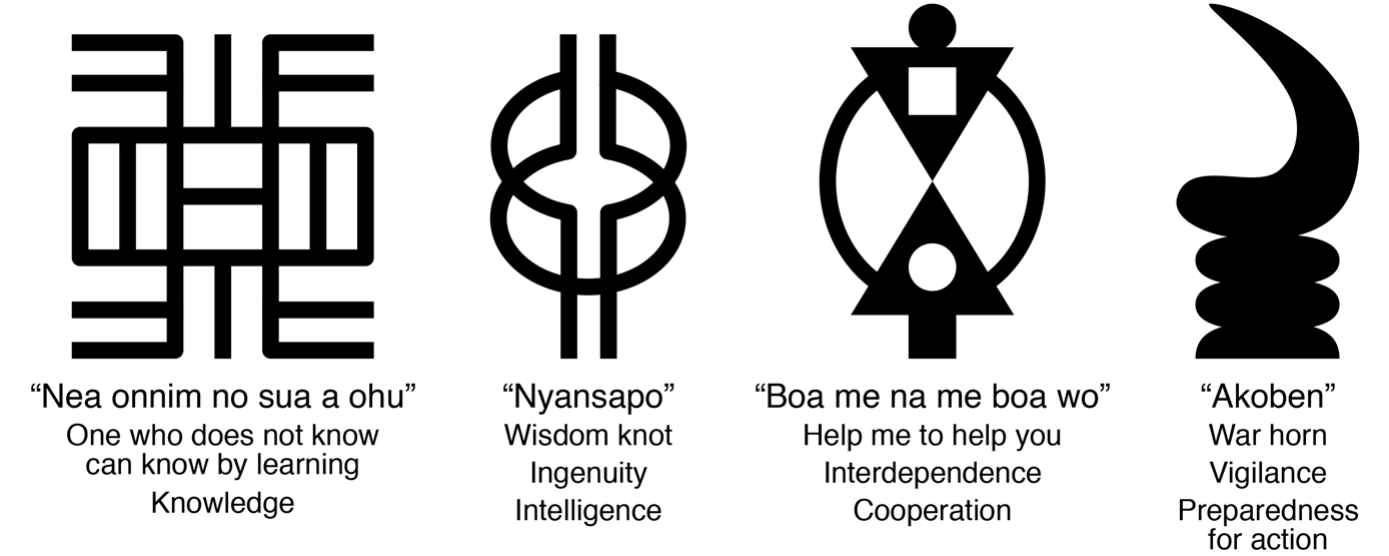
Ghanaian *adinkra* symbols evoking the spirit of the Johns Hopkins–based *JCI* editorial board.

## Version 1 02/01/2022

Electronic publication
